# Enhancing the efficacy of nano-curcumin on cancer cells through mixture design optimization of three emulsifiers

**DOI:** 10.1186/s13065-024-01160-z

**Published:** 2024-03-30

**Authors:** Zahra Sayyar, Hoda Jafarizadeh-Malmiri

**Affiliations:** 1 Department of Chemical Engineering, University of Bonab, Bonab, 55513-95133 Iran; 2 Faculty of Chemical Engineering, Sahand University of Technology, Tabriz, Iran

**Keywords:** Curcumin nanodispersion, Subcritical water, Mixture design, Mixture of emulsifiers, Optimization, Arabic gum

## Abstract

Curcumin, a vital bioactive compound found naturally, has diverse biological applications. However, a major limitation of curcumin is its low bioavailability caused by its limited solubility in water. Hence, it is possible to overcome this problem through preparing oil in water nanodispersion of curcumin that emulsifier can play key role to produce nanodispersion. In the present study, the effect of three emulsifiers of Tween 80, Arabic Gum and Polyethylene glycol on preparing nanodispersions with desirable properties was investigated using subcritical water method and a mixture design. Zeta-potential and particle size of the achieved nanodispersions were taken into account as outcome factors. The optimum values for emulsifiers of Tween 80, Arabic Gum and Polyethylene glycol were obtained as 0.588 g, 0.639 g and 0.273 g, respectively, using the suggested model, so that obtained nanodispersion had minimum particle size (101.89 nm) and maximum zeta-potential (−24.99 mV). In fact, 102.5 nm and − 24.7 mV were obtained from experimental data at these values of emulsifiers. In addition, maximum loading potential (0.199 g/L), efficiency (99.5%), and minimum total curcumin loss (0.5%) were acquired at these optimum values. The results also show that the nanodispersion had a powerful antioxidant activity (65.27%) with extra antibacterial activity in facing with both *E. coli* and *S. aureus* strains. Moreover, curcumin nanodispersion was significantly taken up by HT-29 cells and resulted in the production of oxidative stress in the cells, leading to a decrease in the growth of cancer cells.

## Introduction

Curcumin is a well-known non-polar pigment and a highly hydrophobic molecule with antioxidant properties and various pharmacological effects such as anti-cancer, anti-flammatory, anti-viral, etc. Therefore, it has attracted attention of scientists as a natural component in medicine, beverages, and food [1–3]. Even at high doses of 8–12 g/day, curcumin is highly bioactive with low oral toxicity, making it a potentially safe source of health benefits. However, curcumin’s low water solubility, bioavailability, and instability pose significant challenges for its use in water-based solutions [2, 4, 5].

In response, several nano systems have recently been developed to encapsulate curcumin, such as nano-curcumin liposomal, nano-complexes, colloidal systems, emulsions, nano-emulsions or nanodispersions, and chelation with metals, in order to enhance its bioavailability [6–9]. When compared to other nano-systems, nanodispersions offer a number of benefits, including excellent optical clearness, surface area, and stability from both a physical and chemical perspective. Thermodynamic conditions, i.e., composition, temperature, and pressure, not only affect nanodispersions properties such as particle size distributions, stability and interfacial properties, but also physicochemical properties of bioactive compounds, emulsifiers and their preparation methods and conditions [10–12].

Bioactive compounds can be surrounded by emulsifier molecules that are surface-active substances. There are various food grade emulsifiers in different food products. Emulsifiers are obstacles against coalescence and aggregation of bioactive compounds and protect these compunds from destruction by free radicals. The Gibbse-Marangoni mechanism occurs to prevent aggregation through dynamic surface tension effects and repulsive colloidal interactions. Therefore, selection of emulsifiers is one of the important keys for preparing nanodispersion [13–16]. Small emulsifier molecules can create nanoemulsions with very small particles and physical and chemical instability. Therefore, a mixture of emulsifiers can create nanodispersions with the best physical and chemical stability [16]. Anarjan et al. [17] and Guo et al. [18] showed that a combination of emulsifiers can enhance characteristics of nanodispersions, more effective than one of them, to improve physical and chemical stability during storage.

Therefore, the mixture design experiment is a unique response surface design that can be applied to obtain nanodispersions with improved properties in a multicomponent system. It is used to predict the interaction effects of blended emulsifiers on properties of nanodispersions using linear or non-linear regression models [14, 16].

Also, it is a new technique on the base of subcritical conditions for nanodispersion preparation which is at the same time energy-efficient and has a narrow particle size distribution that can be used to formulate bioactive ingredient, particularly curcumin. Rang of water pressure of 1-221 bar and temperature of 100–324 ºC is generally defined as subcritical water conditions, so that water can preserve its liquidity at these pressure conditions and high temperatures [18–20]. Compared to other methods of nanodispersion preparation, small particles can be produced using subcritical water method and much less amount of emulsifiers. Decreasing the amount of emulsifier required to form a nanodispersion is of great advantages for various reasons like economic, flavour, color and toxicity ones [19–21]. In addition, bioactive components used in other procedures are typically dissolved in a chemical solvent such as ethanol or acetone. Because of this, an additional effort is required to remove the solvent. The solvent, on the other hand, might not be entirely isolated from the nanodispersion products in the end [22, 23]. Curcumin exhibits limited solubility in aqueous solutions. In this study, curcumin nanodispersion was employed to enhance its solubility by increasing its specific surface area. The prepared curcumin nanodispersions demonstrated improved chemical and physical stability compared to pure curcumin, as well as enhanced kinetic stability. The addition of a mixture of emulsifiers further enhanced the physical and chemical stability of the curcumin nanodispersions. Moreover, the presence of this emulsifiers mixture in combination with curcumin resulted in increased pharmacological activity, including improved antibacterial and antioxidant properties, as well as enhanced anticancer effects.

The key objectives of this paper were (i) using subcritical water method to achieve curcumin nanodispersion; (ii) studying the effects of interaction among three selected green emulsifiers of Tween 80, Polyethylene glycol (PEG) and Arabic Gum as stabilizer components on properties of obtained nanodispersions; (iii) optimizing emulsifiers proportions to prepare the curcumin nanodispersion with the highest zeta-potential and the smallest particle size; (iv) investigating the zeta-potential value, polydispersity index (PDI), particle size, morphology, the antibacterial and antioxidant activities and anticancer of the curcumin nanodispersions obtained in this study.

## Materials and methods

### Materials

The curcumin (97% purity), n-Hexane, poly ethylene glycol (P.E.G., 4000 Dalton molecular weight) and Tween 80 used in this study were acquired from Merck Co. (Darmstadt, Germany). Arabic Gum (A.G.) was purchased from Ham food industry group (Ham Co., Tabriz, Iran). 2,2-diphenyl-2-picrylhydrazyl (DPPH), 3-(4,5-dimethylthiazol-2-yl)-2,5-diphenyl-2 H-tetrazolium bromide (MTT), and Dimethyl sulfoxide (DMSO) were bought from Sigma Aldrich (Louis, Missouri, USA) to be utilized in anti-oxidant activity analysis. *Escherichia coli* (PTCC 1270) and *Staphylococcus aureus* (PTCC 1112) were ordered from microbial Persian type culture collection (PTCC, Tehran, Iran). Biolife Company (Milan, Italy) nutrient Count Agar (NCA) was also utilized in this study. The deionized water was used in this study.

### Preparation of samples

Mixtures of Tween 80, PEG and Arabic Gum using the design of augmented simplex-centroid as listed in Table 1, were used for curcumin nanodispersions preparation in subcritical conditions. In this technique, curcumin (about 20 mg) was dissolved in binary of emulsifiers (1.5 g) while the mixture was stirred at 300 revolutions per minute for 15 min. Distilled water (98.5 g) was poured into combination drop-wise that was being stirred for 2 h. The resulting solution was placed in an autoclave at temperatures of 123 ºC and pressure of 1.5 bar for 112 min.

**Table 1:** Central composite design and response variables for production of Curcumin nanodispersions

Run	Tween 80 (X_1_, g)	Arabic Gum (X_2_, g)	PEG (X_3_, g)	Particle size (Y_1_, nm)	Zeta-potential (Y_2_, mV)
1	0.75	0.00	0.75	72.5	−24.70
2	0.00	0.00	1.50	172.0	−27.90
3	0.00	0.75	0.75	496.0	−10.02
4	0.25	0.25	1.00	355.0	−18.78
5	0.50	0.50	0.50	107.0	−24.30
6	1.50	0.00	0.00	16.5	−7.50
7	0.25	1.00	0.25	266.0	−21.60
8	0.00	1.50	0.00	239.7	−17.22
9	0.75	0.75	0.00	304.0	−28.40
10	0.50	0.50	0.50	89.5	−17.50
11	1.00	0.25	0.25	289.7	−28.50
12	0.5	0.5	0.5	96.0	−23.20

### Analysis

#### Mean particle size analysis

Polydispersity index, particles size and zeta potential values of the nanodispersions obtained here were determined using particle size analyzer (Dynamic light scattering, Nanotrac Wave, Microtrac, USA).

#### Colour and turbidity

Curcumin has attracted a great deal of interest and is utilized widely in various formulations of food, drinks and medicine. ColourFlex EZ Spectrophotometer (Hunter Lab. Inc., Reston, VA, USA) was the device used to measure nanodispersions Colour. On the other hand, turbidity analyzer (WTW Co., Turb 555 IR, Weilheim, Germany) was applied to gauge the turbidity of nanodispersions [24, 25].

#### Concentration of curcumin

Equation (1) determines the ratio of weight of curcumin to the volume of curcumin nanodispersions as loading ability (L.A). Nonetheless, the ratio of curcumin in the nanodispersion obtained to added amount of initial curcumin is defined as loading efficiency (L.E.). The percentage of loaded curcumin was measured using Eq. (2) [6].

1 $$L.A. = \frac{{Weight\,of\,Curcumin}}{{Volume\,of\,Nanodispersion}}$$

2 $$L.E.(\% ) = \frac{{{A_2}}}{{{A_1}}} \times 100$$

Where A_1_ is amount of initial curcumin and A_2_ is the amount of curcumin in samples calculated from UV-Visible (Perkin Elmer Co., 250–800 nm, Rodgau, Germany). Concentration of curcumin was evaluated using provided standard curve. Standard curve was determined from our previous work [26]. The curcumin loss are Eq. (3) is used to calculate:

3 $${\text{Curcumin loss}}\left( \% \right)=\frac{{{C_0} - {C_1}}}{{{C_0}}}$$

Where C_0_ is initial concentration of curcumin (0.2 g/L) and C_1_ is the curcumin concentration in the obtained nanodispersion [27, 28]. Concentration of curcumin in nanodispersions was calculated through adding curcumin nanodispersions (3 mL) to n-hexane (3 mL) and centrifuging the resulting mixture at 1500 rpm using a laboratory centrifuge, at room temperature, for 15 min (Hettich Zentrifugen, EBA 20, Tuttlingen, Germany). During this stage, a nanodispersion created a clear top layer (n-Hexane) and an opaque sediment layer at the bottom (curcumin), with a creamy or oily layer in the middle (mixture of emulsifier). After separating the top n-Hexane layer, the extraction process was repeated using the bottom layer under the same conditions. Finally, the concentration of curcumin in the bottom layer was measured using a UV-Vis spectrophotometer [29].

#### Rheological attributes

The apparent viscosity and shear stress were measured in rotational mode using a rheometer (Anton Paar, MCR 92, Graz, Austria) with a ball-bearing motor. In fact, nanodispersion behavior was demonstrated through values of µ and n acquired from the fitting plot of logarithm shear rate against $\dot{\gamma}$ logarithm shear stress τ indicates [30].

#### Antioxidant activity

Brand-Williams [31] modified method to evaluate antioxidant activity of the bioactive components. In this study, 100 µL of the sample were mixed with methanol (5 mL, 50%) solution including DPPH (1mM) to determine the DPPH scavenging capacity. The control sample was created by combining pure DPPH with methanol in a 1:1 ratio, followed by thorough stirring and incubation in a dark area at 27 ˚C for 30 min. The maximum absorbance (517 nm) of the solutions and their blank were noted. Then the inhabitation percentage was obtained [32].

#### Antibacterial activity

To evaluate the effectiveness of curcumin nanodispersion beside Gram-positive (S. aureus) and Gram-negative (E. coli) bacteria, the well diffusion method devised by Torabfam et al. [33] was utilized. Plates of PCA culture medium (90 mm in diameter) were inoculated with bacterial suspensions containing 1.5 × 10^8^ colony-forming units per milliliter of obtained suspensions. Each of the 5 mm openings in the inoculated culture medium was filled with 10 mL of curcumin nanodispersion. The dishes were then incubated at 37 °C for 24 h. Indicative of the nanodispersion’s antibacterial activity was the formation of a clear zone around the perforations on the plate.

#### Cytotoxicity

Based on our prior study, we conducted an MTT test to evaluate the cytotoxic impact of pure curcumin and curcumin nanodispersion [3, 13]. To carry out the test, we seeded 10,000 of L929 and HT-29 cells onto a 96 well and incubated them overnight. Then, we exposed them to 2% samples and kept them in an incubator for 24, 48, and 72 h (at 37 °C incubator with 5% CO_2_ and 100% humidity). After incubating the samples with 100 µL of MTT solution (5 mg MTT/mL PBS) for 4 h, we centrifuged them at 3000 rpm for 3 min in order to remove any unreacted MTT solution. Subsequently, 100 µL of DMSO was poured into the wells in order to dissolve the formazan crystals that were generated by live cells. After shaking the samples for ten minutes, we used an ELISA microplate reader (Biochrom Asys Expert 96, MA, USA) to determine the absorbance of the formazan dye at 570 nm. This was done after first agitating the samples for ten minutes. We performed the experiment three times to ensure the results’ repeatability.

#### Structure of samples

The particle size and shape and morphology of sample were investigated using Transmission electron microscopy (TEM, CM120, Philips, Amsterdam, Netherlands) with 120 kV voltage.

### Experimental design

Compared to other experimentation methodologies, the mixture design has several advantages. In order to acquire a product with desired properties, proportions of emulsifiers can be optimized by the mixture design. Therefore, 12 experiments were run (Table 1), data was analyzed, graphs were plotted, and the optimum amount of emulsifiers were obtained by Minitab v.18 statistical software package (Minitab Inc., PA, USA). All tests were performed in one day by applying an augmented simplex-centroid mixture design, so that the effects of interaction between emulsifiers’ components can be investigated on average particle size (Y_1_) and zeta-potential (Y_2_) of the curcumin nanodispersion. The experimental data contains different proportions of emulsifiers of X_1_ (Tween 80), X_2_ (Arabic Gum) and X_3_ (PEG) between 0 and 1.5 (ƩX_i_ = 1.5). Three repetitions of the center point were made to estimate pure error. Mixture design was applied to conclude the importance of the model terms, and quality of the fitted models on the base of determination coefficient (R^2^). The special cubic models as Eq. (4) were applied to correlate size and zeta-potential of curcumin nanodispersion particles to estimate values of emulsifiers.

4 $$\begin{aligned} {Y_i} & ={a_1}{X_1}+{a_2}{X_2}+{a_3}{X_3}+{a_{12}}{X_1}{X_2} \\ & \quad +{a_{13}}{X_1}{X_3}+{a_{23}}{X_2}{X_3}+{a_{123}}{X_1}{X_2}{X_3} \\ \end{aligned}$$

Where Y_i_ is the particle size or zeta-potential, and *a*_*i*_, *a*_*ij*_ and *a*_*ijk*_ are the regression coefficients for each linear, binary and ternary effects of interaction terms, respectively. With the help of the coefficient of determination (R^2^) and the modified coefficient of determination (R^2^-adj), the adequacy of the model was investigated. The model that was developed thereafter underwent an analysis of variance (ANOVA), and the results were interpreted in terms of p-value. P-values lower than 0.05 were regarded as statistically significant by the researchers. Fitted equations were utilized to generate contour plots in order to predict how a response was associated to three emulsifier component.

#### Optimization of operational condition

To achieve optimal values, graphical optimizations were applied for different proportions of components (X_1_, X_2_ and X_3_) along with numerical multiple response and suitable response variables (Y_1_ and Y_2_). Optimal proportions of components were obtained by the mixture design optimizer in the Minitab software. Finally, three extra approval tests were conducted at obtained optimum proportions of components to validate experimental data. This can be used as a proper technique to optimize the proportions of emulsifiers [33, 34]. Decency of the mixture design models was corroborated by matching data from experimental with the data obtained from final models.

### Statistical analysis

Preparation of the samples and all analyses assessment were accomplished in three replicates. Analysis of variance and Tukey’s Comparison test, based on Minitab statistical software (v.16, Minitab Inc., PA, USA), were used in data interoperation and comparison, respectively with significance level of 95% (p < 0.05).

## Results and discussions

The application of nanotechnology in agriculture has had a significant effect on various aspects of farming. One of its benefits is the use of natural nanomaterials, which can improve plant growth, resist external factors, increase the production of active compounds and secondary metabolites, as well as enhance crop yields and physiological pathways. In this study, biological properties of curcumin were investigated.

### Fitting the initial mixture design model

Final models were produced based on special cubic model and the experimental data achieved for particle size and zeta-potential (Table 1) using multiple regression analysis in order to investigate the mixture of emulsifiers. Table 2 shows regression coefficients such as *a*_*1*_, *a*_*2*_, *a*_*3*_, *a*_*12*_, *a*_*13*_, *a*_*23*_, *a*_*123*_, R^2^, R^2^-adj, p-value and F-value terms for models.

**Table 2:** Regression coefficients, R^2^, adjusted R^2^ and probability values for the final reduced models

Regression coefficient	Particle size (Y_1_, nm)	Zeta-potential (Y_2_, mV)
*a*_*1*_	23	−4.98
*a*_*2*_	255	−11.55
*a*_*3*_	165	−18.62
*a*_*12*_	749	−28.57
*a*_*13*_	3171	13.69
*a*_*23*_	1178	22.17
*a*_*123*_	−16,234	−48.14
R^2^ (%)	96.4	93.8
R^2^_−_adj (%)	91.01	81.41
F-value	44.42	0.91
Lack-of-Fit (p-value)	0.82	0.77

In addition, the p-values and the F-values of the independent variables in the models that were gathered are provided in Table 3. These relatively high values for R^2^ and R^2^-adj confirm overall model performance; therefore, their values for particle size (96.4% and 91.01%, respectively) and zeta-potential (93.8% and 81.41%, respectively) of the obtained curcumin nanodispersion verified the Suitability of the suggested models.

**Table 3:** Significance probability (p-value and F-value) of regression coefficients in the second-order polynomial models

	Main effects		Quadratic effects	
	X_1_X_2_	X_1_X_3_	X_2_X_3_	X_1_X_2_X_3_
**Particle size (Y**_**1**,_ **nm)**				
p-value	0.022	0.006	0.005	0.001
F-value	13.34	29.58	33.03	61.37
**Zeta-potential (Y**_**2**_, **mV)**				
p-value	0.022	0.061	0.042	0.043
F-value	19.25	0.32	11.69	0.81

The results presented in Table 3 show that the interaction effects of emulsifier compounds of coefficients of regression in ultimate special cubic polynomial models significantly affected (*p* < 0.05) curcumin nanodispersion’s particles size and zeta-potential. Cubic model was applied to predict the effect of individual emulsifier, binary and ternary interactions among them on particles size or zeta-potential by significant interaction terms.

### Effect of the emulsifier mixture on particles size

The experimental data on the particles size of obtained samples ranged 16.5–496 nm (Table 1). According to these findings, an appropriate range for nanodispersion was found. In addition, they proved that the curcumin nanodispersion that was generated for this study could be simply formed without the need of any additional chemical solvents. This was an important finding. These results confirmed that Arabic Gum had the most significant effect (*a*_*2*_ > *a*_*1*_ and *a*_*3*_) on the particles size. The final model of regression fitted for particles size contains all terms of special cubic model. All the positive effects of emulsifiers (Tables 2 and 3) in the final model for particles size exhibited that three emulsifiers synergically act to each other. Therefore, using a blend of emulsifiers could increase curcumin nanodispersion particle size. Nonetheless, negative ternary blend of all three emulsifier components (X_1_×X_2_×X_3_) indicated an antagonistic effect. These effects could reduce curcumin nanodispersion particles size. Therefore, applying all three emulsifier components can prepare a curcumin nanodispersion with small particles size.

**Fig. 1:**
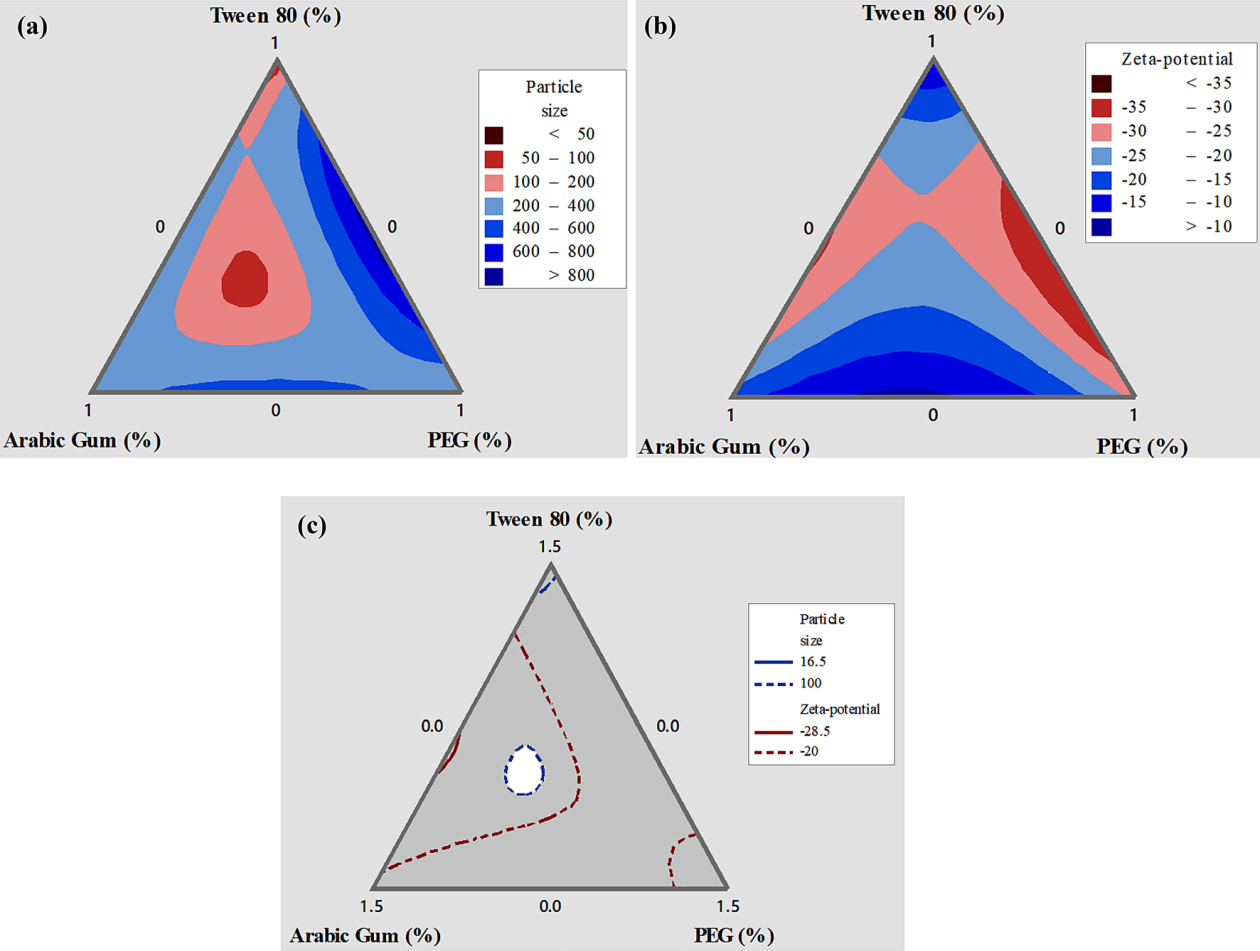
Surface contour of estimated **a**) average particle size (nm), **b**) zeta-potential (mV) as a function of used emulsifier components according to the special cubic model and **c**) Plot of overlaid contour of emulsifier components to get the most desirable Curcumin nanodispersions

On the other hand, p-values of the interactions demonstrated that, due to small p-value, ternary blend effect of emulsifier on particle size is more important than other interactions. Figure 1a shows these effects in the contour plots. The binary mixture of emulsifiers also established a significant decline in mean curcumin nanodispersion particles size in subcritical water conditions. These results demonstrated that, the mixture of emulsifiers affected the particles size because of its effect on the rate of crystallization. Thus, the smallest particles could be yielded in certain optimum proportion of emulsifiers and their size and shape were controlled. Anarjan et al. [14] showed that using small emulsifier’s molecule can prepare nanodispersions with small particle size, that confirms our results as well.

### Effect of the emulsifier mixture on zeta-potential

The experimental data on zeta-potential for the obtained samples were ranged − 28.5 to −10.2 mV (Table 1). A special cubic model as a function of emulsifier mixture was used to predict zeta-potential due to P < 0.05 and R^2^ = 93.8%, as presented in Tables 2 and 3. As shown in Table 1, ternary blend of all three emulsifier components (X_1_×X_2_×X_3_) has the most significant effect on zeta-potential due to high regression coefficient. Moreover, binary interactions of PEG and Tween 80 (X_1_×X_3_) were deleted from the final model of regression fitted for zeta-potential due to high p-value (p>0.05). However, since the binary interaction coefficient of Tween 80 and Arabic Gum is negative and high, these two components are antagonistic to each other and this, in turn, increases zeta-potential of curcumin nanodispersion. The effect of the emulsifiers blend on zeta-potential of curcumin nanodispersion is shown in Fig. 1b. Commonly, applying all three emulsifiers can induce high zeta-potential in the obtained nanoparticles. A high zeta potential value is indicative of greater stability, as it signifies a strong repulsive force between droplets, preventing them from flocculating or coming into close contact. Conversely, a low zeta potential value implies lower stability, as the weak repulsive force between droplets allows for the possibility of flocculation and close contact. To prevent coalescence and maintain the stability of nanodispersion, it is important to maintain an appropriate electrostatic repulsion between droplets, typically indicated by a high zeta potential value. The findings of this study revealed significant differences (*p* < 0.05) among the samples, indicating that the concentrations of emulsifiers had an impact on the stability of the nanodispersions. The samples with the highest zeta potential values exhibited the strongest electrostatic repulsion between droplets, resulting in a more stable nanodispersion.

### Optimization of proportions of emulsifiers for sample

Optimum proportions of emulsifiers for preparing curcumin nanodispersion can be reached when the obtained samples have a small particles size and high zeta-potential. Graphical optimization for the proportions at suitable value of emulsifiers can be seen in Fig. 1c. The white colored area displays optimum emulsifier proportions in samples based on an overlaid contour plot. Curcumin nanodispersion obtained with Tween 80 (0.588 g), Arabic Gum (0.639 g) and PEG (0.273 g) had the minimum particles size (101.89 nm) and maximum zeta-potential (−24.99 mV) which confirmed the results of numerical multiple optimizations. To test the correctness of the discovered and fitted models via mixture design, three derived curcumin nanodispersions were evaluated in terms of particle size and zeta potential. The experimental data for particle size and zeta-potential of curcumin nanodispersion indicated that no differences of any significance were found comparing experimental and predicted values of the obtained curcumin nanodispersion. Therefore, these results confirm the adequacy of the models obtained by mixture design.

### Particles size of sample obtained at optimum proportions of emulsifiers

**Fig. 2:**
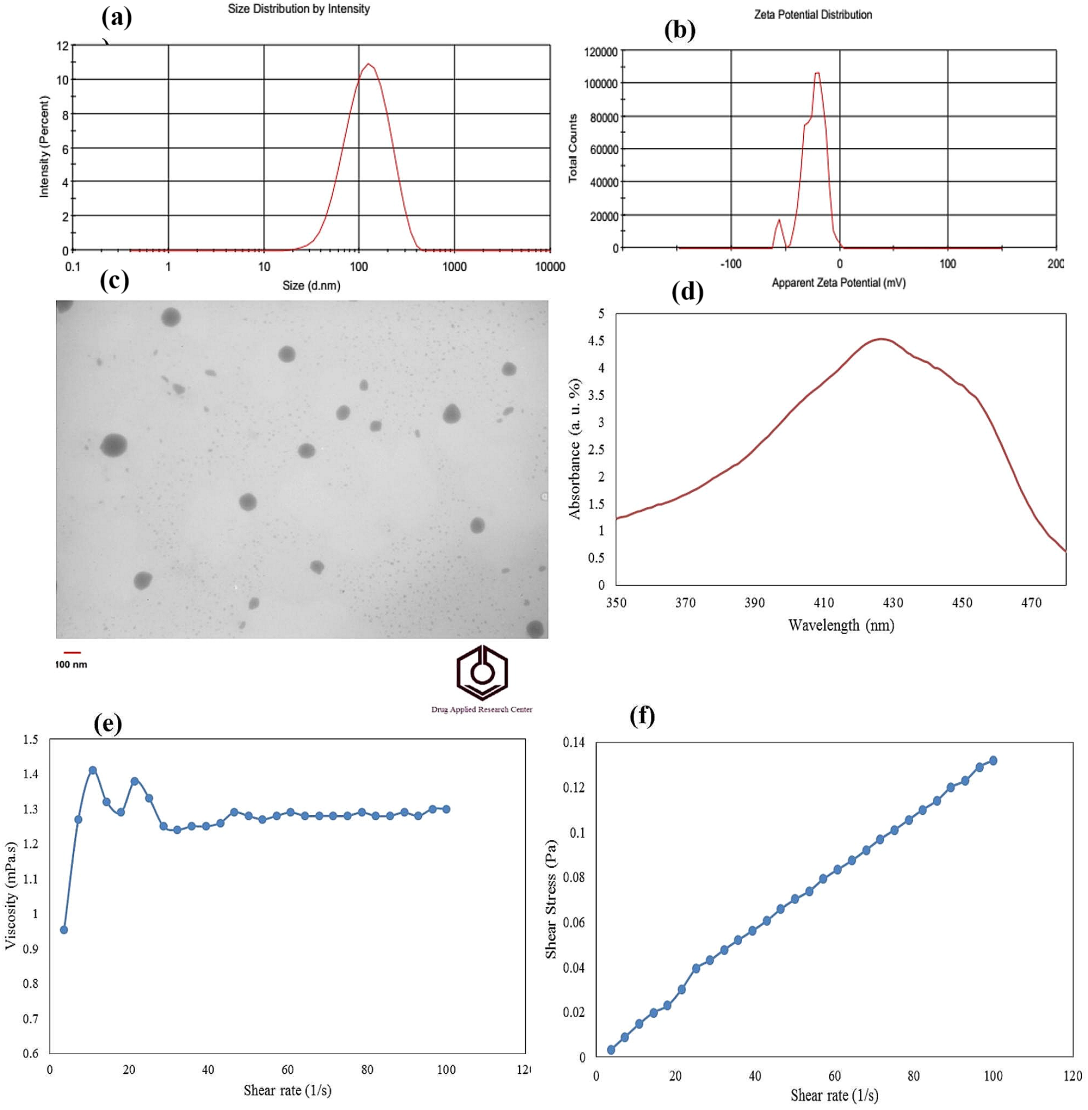
Plot of **a**) particle size distribution, **b**) zeta-potential distribution, **c**) TEM image, **d**) UV–Vis spectrometry analysis, **e**) Flow profiles viscosity versus shear rate and **f**) shear stress versus shear rate measured at 25 ˚C of the prepared Curcumin nanodispersion at obtained optimum of emulsifier components

Formulation development plays a crucial role in the production of nanodispersions, as it enables the achievement of specific criteria such as a small particle size within the nanometer range (and a low Polydispersity Index (PDI). Furthermore, the particle size is of great importance as it not only determines the stability and appearance of the nanodispersion but also influences its bioavailability and the texture of the final product. According to DLS results, the curcumin nanodispersion obtained at optimum proportions of emulsifiers had mean particle size value equal to 102.5 nm, PDI value equal to 0.215 and zeta potential value equal to −24.7 mV. Figure 2a shows the particles size distribution of curcumin nanodispersion obtained at optimum proportions of emulsifiers. It also displays that all the particles ranged 10–110 nm. As shown in Fig. 2b, distribution curve of zeta-potential of the curcumin nanodispersion obtained at optimum proportions demonstrated that stability increased as zeta values of curcumin nanodispersion obtained at optimum operational condition increases. The findings showed that the predicted values were quite close to the experimental values. Thus, suggested special cubic models were suitable to predict the mean particles size and zeta-potential as functions of emulsifier component proportions.

### Morphology of the sample obtained at optimum proportions of emulsifiers

The morphology and size distribution of samples obtained with proportions of emulsifiers evaluated by a TEM was shown in Fig. 2c. The results revealed dispersion of spherical curcumin nanoparticles with average particle size of 50–100 nm. As can be easily seen, sample at optimum proportion of emulsifiers had sphere-shaped and harmonic morphology. In fact, the small particle size showed the sample with minimum surface energy. Furthermore, zeta-potential’s high value in curcumin nanodispersion increased thermodynamic, physical and chemical stability.

### Turbidity and colour of sample obtained at optimum proportions of emulsifiers

Curcumin nanodispersion obtained using binary of emulsifiers and through subcritical water method at optimum operational condition was visually transparent. The clear appearance showed very weak light scattering because of the smaller particles size than optical wavelength of the visible spectrum. As a result, it is necessary to take readings of the samples’ colors as well as their turbidities. Whiteness index (WI) is a metric that is used to express the optical characteristics of nanodispersion.

The results showed that the obtained sample at optimum proportions of emulsifiers had a yellow color with positive b^*^ value (b^*^= 3.67). Also, a* value were close to zero representing neutral and tending to red color (a^*^=0.299). The sample had the lowest L^*^ value (3.19) with more transparency. Curcumin nanodispersion prepared at optimum proportions of emulsifiers having least particle size displayed lowest L* value indicating minimum whiteness index and turbidity. A main feature of nanodispersions is low turbidity of the colloidal solutions. It can be concluded that sample at optimum proportions of emulsifiers had maximum transparency (WI = 3.12) and minimum turbidity value (0.084 NTU). The whiteness index results can be explained by particles size of sample at optimum proportions of emulsifiers; therefore, the smaller the particles of the nanodispersion, the lower its WI. As the particle size of the nanodispersions increases and approaches size of wavelength of light, scattering of light increases as well causing nanodispersion to appear turbid and white.

### L.A. and L.E. of curcumin content at optimum proportions of emulsifiers

UV–Vis analysis of sample at optimum proportions of emulsifiers with their maximum absorbance peak at 420–435 nm is shown in Fig. 2d. This revealed curcumin L.A. (g/L), L.E. (%) and curcumin loss (%) in study samples. The results demonstrated that using binary of emulsifiers can yield nanodispersion with maximum L.A (0.199 g/L) and L.E. (99.5%); however, curcumin nanodispersions had minimum total curcumin loss (0.5%). Moreover, these results show that water in subcritical conditions can act as polar liquid solvents, for example, methanol, ethanol, and acetone. In fact, solubility of curcumin increases because the hydrogen bond between water molecules becomes very weak. Comparing others results of L.E. value, formulation of curcumin under subcritical conditions, using binary of emulsifiers increased the L.E [6, 35]. Curcumin obtained with subcritical water and a mixture of emulsifiers was as soluble as in organic solvents, demonstrating the superiority of this method in comparison to others.

The stability of colloidal systems is crucial for their utilization in various industries such as food, beverage, cosmetics, and pharmaceuticals. In this study, the physico-chemical stability of curcumin nanodispersions was evaluated by monitoring the average size of the particles as an indicator of physical stability. Additionally, the concentration of Curcumin was assessed by storing the samples in a dark environment at both 4 and 25 °C for a duration of 3 months to evaluate their chemical stability.

The physical and chemical stabilities of the Curcumin nanodispersions were assessed by monitoring changes in particle size and the loss of Curcumin during storage. Table 4 presents the particle sizes of the nanodispersions at room temperature (25 ºC) and refrigerator temperature (4 ºC) over time. The results revealed that the nanodispersions stored at refrigerator temperature exhibited greater physical stability compared to those stored at room temperature. This suggests that the movement of particles was slower at the lower temperature, contributing to enhanced stability.

**Table 4:** Particle size and curcumin loss for the prepared nanodispersions

Storage time (Day)	Particle size		Curcumin loss%	
	25 ºC	4 ºC	25 ºC	4 ºC
0	102.5	102.5	0.50	0.50
30	103.4	102.5	0.58	0.53
60	104.1	102.8	0.63	0.55
90	104.5	102.5	0.69	0.60

### Rheological attributes of the sample obtained at optimum proportions of emulsifiers

This property is important for various processes involved in food processing, such as mixing, pouring and pumping where different operative shear rates are applied. The apparent viscosity as an essential factor against shear rate of the nanodispersion obtained at optimum proportions of emulsifiers is displayed in Fig. 2e. An example of Newtonian behavior was seen in these samples as a result of the fact that the apparent viscosity of the sample rose as the shear rates increased.

According to Fig. 2f, to fit the data with a high correlation (R^2^ = 0.99), power law model can be applied when consistency index (µ) is 1.038 mPa.s and flow behavior index (n) is 1.075. According to power law index, viscosity of nanodispersion obtained by a blend of emulsifiers was not very high. Low viscosity of nanodispersion obtained using a blend of emulsifiers are appropriately applicable in food and drug industries producing beverages and oral drugs requiring low viscosity. Mean particles size significantly decreases due to presence of the nonionic emulsifiers such as Tween 80, which droplet disruption had happened at subcritical water condition to make the viscosity of the two phases aqueous and oil the same.

### Antioxidant assay of the sample obtained at optimum proportions of emulsifiers

Anti-oxidants can donate hydrogen from DPPH because of de-colorization of the DPPH in the presence of anti-oxidants and deep purple color at 517 nm [36]. The antioxidant activity of the sample obtained at optimum proportions of emulsifiers was acquired. The results indicated that the sample had a powerful antioxidant activity (65.27%). They also discovered that the strategies that were utilized had an effect on the nanodispersions’ capacity to scavenge free radicals. It can be established that the results obtained in the present work are in the usual retention range of curcumin encapsulated in nanodispersion at optimum proportions of emulsifiers with applications for the food industry.

### Antibacterial activity of the sample obtained at optimum proportions of emulsifiers

**Fig. 3:**
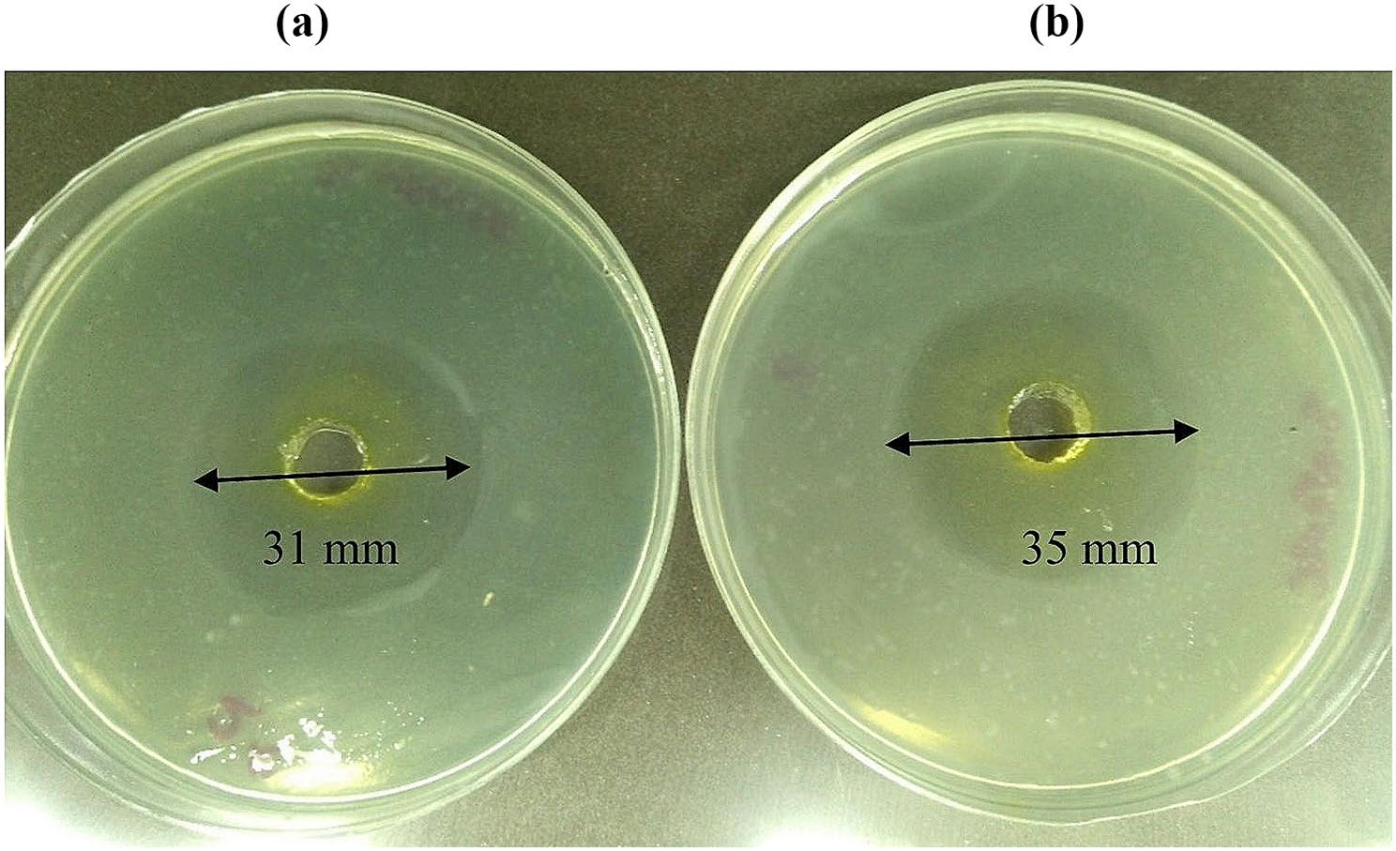
Created zones of inhibition of the prepared Curcumin nanodispersion at obtained optimum of emulsifier components **a**) E. coli and **b**) S. aureus incubated at 37 ºC for 24 h

Antibacterial activity (clear zone) of the sample obtained at optimum proportions of emulsifiers is shown in Fig. 3. Findings of this study revealed that the nanodispersion obtained at optimum proportions had sufficient antibacterial activity against both *S. aureus* (35 mm) and *E. coli* (31 mm). Though, according to findings of the study, the antibacterial activity of obtained sample was lower against *E. coli* compared to *S. aureus*. Our previous study had also proved the same. That study had shown similar effects of bacteria.

### MTT assay

**Fig. 4:**
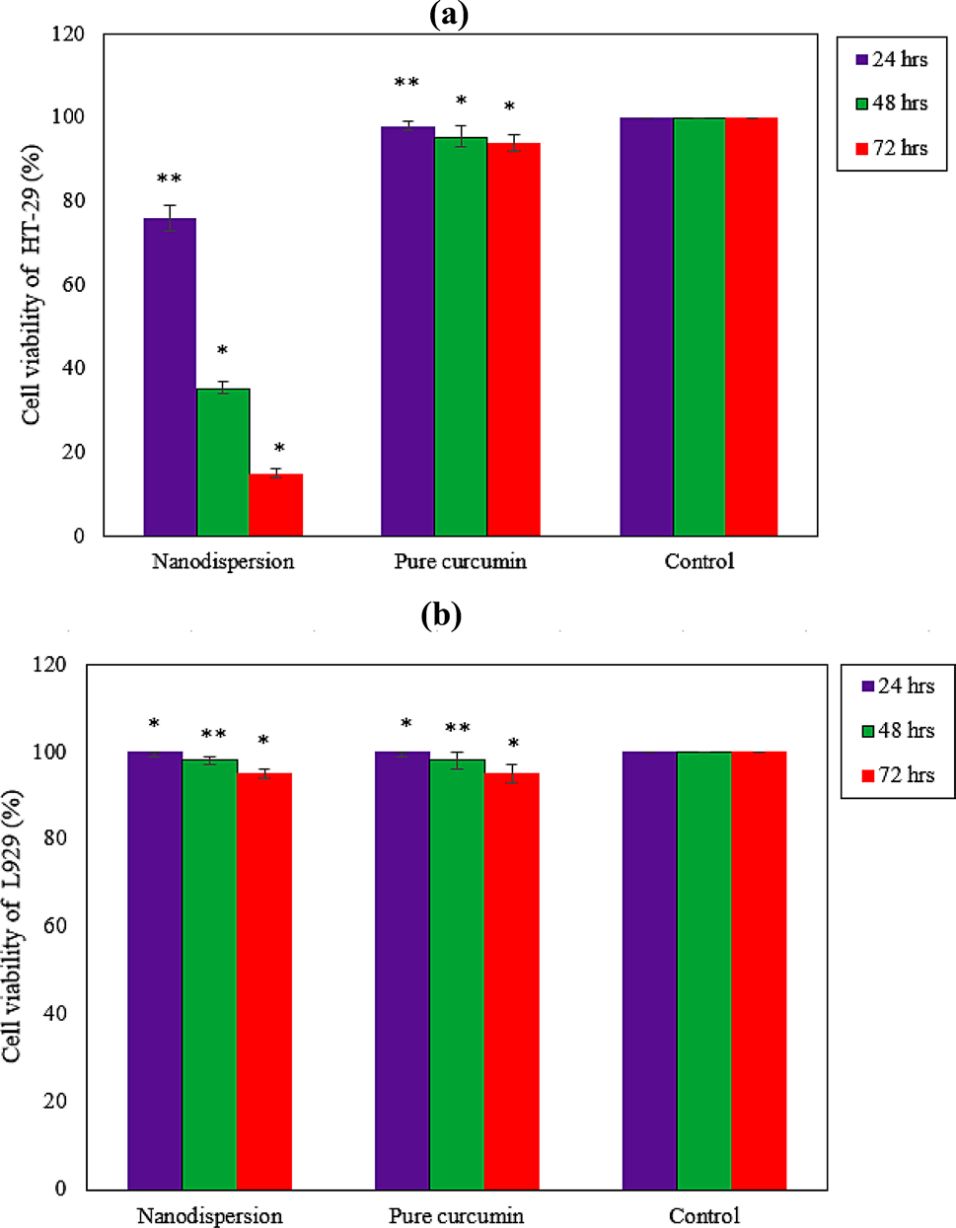
Cell viability percentage for the (**a**) cancer cells (HT-29) and (**b**) normal human fibroblastic cells (L929) for 24, 48, and 72 h incubation with pure Curcumin and prepared nanodispersions at optimum proportions of emulsifiers (mean values ± standard deviation of triplicate assays from three independent experiments). *, ** Significantly different from control at *p* < 0.01, *p* < 0.05, respectively

Figure 4 displays the cell viability percentages for cancer cells (HT-29) and normal human fibroblastic cells (L929) when treated with pure curcumin and obtained curcumin nanodispersions at optimum proportions of emulsifiers. After 72 h of incubation, the viability percentage of HT-29 cells subjected to pure curcumin and obtained curcumin nanodispersions at optimum proportions of emulsifiers were 94% and 15%, respectively. This demonstrates that the viability of HT-29 cells was dramatically decreased by the obtained curcumin nanodispersion at the ideal emulsifier proportions. Moreover, the results revealed that the obtained sample was most effective in inhibiting the growth of HT-29 cancer cells. The toxicity of obtained curcumin nanodispersion was found to be higher on cancer cells than pure curcumin because the nanoparticles of curcumin were better absorbed by the cells. The results obtained from the study showed that curcumin nanodispersion was not harmful to normal L929 cells. The outcomes suggested that curcumin nanodispersion was significantly taken up by HT-29 cells and resulted in the production of oxidative stress in the cells, leading to a decrease in the growth of cancer cells.

## Conclusion

The curcumin nanodispersions containing selected emulsifiers as Tween 80, Arabic Gum and PEG were obtained using subcritical water conditions. The mixture design as simplex-centroid mixture was used to optimize emulsifier value to obtain curcumin nanoparticles with maximum zeta-potential and minimum particle size. The results showed that the suggested model was a suitable model because of the most desirable physicochemical characteristics by comparing experimental values and the predicted values. The optimum values for emulsifiers of Tween 80, Arabic Gum and Polyethylene glycol were obtained as 0.588 g, 0.639 g and 0.273 g, respectively, that created minimum particles size (101.89 nm) and maximum zeta-potential (-24.99 mV) in nanodispersion. The antioxidant result indicated that the sample had a powerful antioxidant activity (65.27%). According to power law index (*n* = 1.075), viscosity of nanodispersion obtained by a blend of emulsifiers was very low. The largest growth inhibition zone diameter for both Gram-positive (35 mm) and Gram-negative bacteria (31 mm) was obtained. Moreover, the viability percentage of HT-29 cells subjected to pure curcumin and obtained curcumin nanodispersions at optimum proportions of emulsifiers were 94% and 15%, respectively. It implies that improving the processing parameters is required to achieve nanodispersion that has superior biological properties and can be employed in agriculture, cosmetics and pharmaceuticals.

## Data Availability

All data generated or analyzed during this study are included in this published article.
